# Female Choice Reveals Terminal Investment in Male Mealworm Beetles, *Tenebrio molitor*, after a Repeated Activation of the Immune System

**DOI:** 10.1673/031.011.5601

**Published:** 2011-05-02

**Authors:** I Krams, J Daukšte, I Kivleniece, T Krama, MJ Rantala, G Ramey, L Šauša

**Affiliations:** ^1^ Department of Biology, University of Turku, FIN-20024 Turku, Finland; ^2^ Institute of Systematic Biology, University of Daugavpils, LV-5401 Daugavpils, Latvia; ^3^ Department of Life Sciences, Anglia Ruskin University, Cambridge CB 1PT, UK

**Keywords:** immune challenge, sexual selection, terminal investment

## Abstract

Increasing evidence suggests that secondary sexual traits reflect immunocompetence of males in many animal species. This study experimentally investigated whether a parasite-like immunological challenge via a nylon implant affects sexual attractiveness of males in *Tenebrio molitor* L. (Coleoptera: Tenebrionidae) Although a single immunological challenge significantly reduced sexual attractiveness and locomotor activity of males, it had no adverse effect on their survival. A second immune challenge of the same males increased their attractiveness. However, it was found that the repeated challenge significantly reduced locomotor activity of males and caused higher mortality. This result indicates terminal investment on sexual signaling, which is supposedly based on a trade-off between pheromone production and energy expenditures needed for such activities as recovery of immune system and locomotor activity. When the third implantation was carried out in the same group of males, melanization of nylon implants was found to be lower in more attractive than in less attractive males. This suggests that males that became sexually attractive after the second immune challenge did not invest in recovery of their immune system.

## Introduction

When parasites challenge an organism's immune system they reduce its fitness by decreasing survival or reproductive success. In response, host organisms have developed various defence strategies and mechanisms. The theory of ecological immunology predicts that investment in immune defence is balanced against investment in other important traits, such as sexual signaling or developmental rate (e.g., Ahtiainen et al. 2005; Rantala and Roff 2005; Folstad et al. 1989; Sheldon and Verhulst 1996). This notion suggests that, in considering evolutionary trade-offs, immune defence can be treated similarly to other life history traits (Zuk and Stoehr 2002).

Organisms cannot simultaneously maximize all life-history traits that promote survival, growth, reproduction, and health (Stearns 1992). Thus, organisms must distribute their resources among competing systems to maximize lifetime reproductive success. One prediction from life-history theory, termed the ‘terminal investment hypothesis’, suggests that animals should invest more in current reproductive output if the chance of surviving to next reproduction is low (Clutton-Brock 1984). Although examples of this phenomenon have been documented in wild animals, the underlying mechanisms producing these outcomes remain largely unstudied. It has been shown that males have higher fitness payoffs from such terminal investment compared to females (Bateman 1948; Sadd et al. 2006), because males may increase their fitness by copulating with many females. Immune responses against parasite attacks often result in parasites dying without harming the host individual (Yourth et al. 2001, 2002). However, repeated attacks by parasites may serve as a reliable signal of the reduced probability of reproduction and survival in the future.

Insect immunity is characterized by an inducible expression of a large array of antimicrobial peptides and the constitutive melanization—encapsulation response (Siva-Jothy et al. 2005; Schulenburg et al. 2009). Encapsulation is a nonspecific, constitutive, cellular response through which insects defend themselves against multicellular pathogens, such as fungi and parasitoids (Yourth et al. 2001, 2002). Encapsulation is an immune response in which some hemocytes recognize an object as foreign and cause other hemocytes to aggregate and form a capsule. A cascade of biochemical reactions leads to the deposition of melanin and the hardening of the capsule (Gillespie et al. 1997). The enclosed intruder dies from suffocation or from the release of necrotizing compounds (Nappi et al. 1995; Yourth et al. 2001, 2002).

In invertebrates, one way to assay this reaction is to measure the magnitude of the encapsulation response to a novel and standardized antigen, such as a nylon monofilament, which is used as a synthetic parasite (e.g., Köning and Schmid-Hempel 1995; Rantala et al. 2000, 2002, 2003a,b; Koskimäki et al. 2004; Vainio et al. 2004; Ahtiainen et al. 2004, 2005). It has been shown that the ability to encapsulate nylon monofilament is strongly related to the ability to encapsulate a real pathogen (Rantala and Roff 2007). It is widely appreciated that beetle immune systems respond to these types of inserts similarly to an invasion by a parasitic or foreign body and attempt to encapsulate it. Specifically, phenoloxidase enzyme production is activated, which leads to melanization of the formed capsule (Ratcliffe et al. 1985). The resulting coating formed by cellular materials and chemical deposits on the insert darkens its color and the extent of darkening correlates to the level of immune system response. In other insects, the darkening correlates with some measures of immunity, such as phenyloxidase cascade (Rantala et al. 2000, 2002; Siva-Jothy 2000; Rantala et al. 2007). The ability to encapsulate a synthetic substrate was shown to be positively related to encapsulation of parasites (Paskewitz and Riehle 1994; Gorman et al. 1998) and to the ability to resist an entomopathogenic fungal disease in moths (Rantala and Roff 2007). Therefore, higher levels of melanization of the inserts indicates increased levels of immune system activity and response (Yourth et al. 2001, 2002).

**Figure 1.**
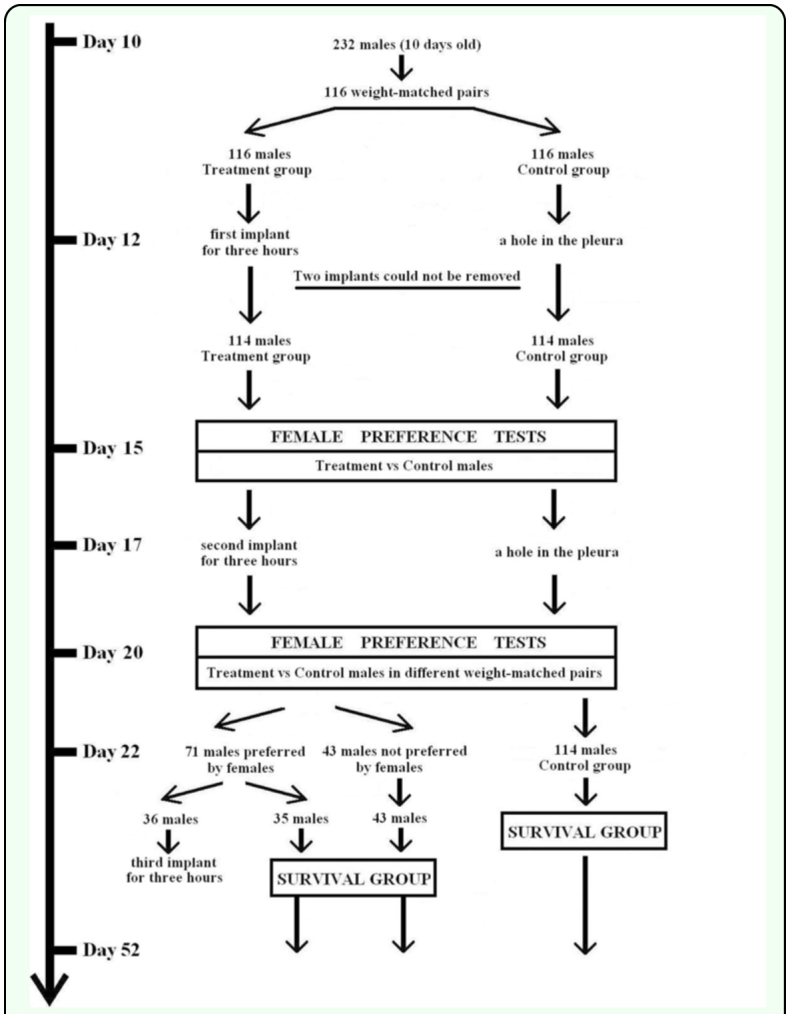
The experimental protocol used to prepare male mealworm beetles of control and experimental groups for female preference and survival tests. High quality figures are available online.

Recently, Sadd et al. (2006) found that in male mealworm beetle activation of the immune system by a nylon monofilament reduced survival in male mealworm beetles but increased attractiveness of the male sex pheromone, suggesting terminal investment. However, with substantially larger sample size and with various immune challenge methods, Vainikka et al. (2007) found neither a survival cost of the activation of the immune system nor an effect on the attractiveness of the pheromone. Since male attractiveness is dependent on individual-level investment decisions reflecting reproductive effort, it may change dynamically over the course of a male's life (August, 1971; Hurd & Parry, 1991; Sadd et al. 2006).

In this study, we tested whether immunological challenge to adult *Tenebrio molitor* L. (Coleoptera: Tenebrionidae) males via a nylon implant would affect their attractiveness to females. Three experiments were conducted where the male immune system was challenged and their attractiveness was measured in two mate choice trials (Figure 1). First, female choice was examined between males that had received an implant and control males that had not been experimentally treated. It was predicted that, for cases where implantation did not cause any acute adverse effects, immune challenge would decrease the attractiveness of males because of a trade-off between immune function and sexual attractiveness of males. Repeated implantations were done to evaluate whether after simulated repeated parasite attack male mealworm beetles exhibited increased investment in sexual traits consistent with terminal investment hypothesis (Sadd et al. 2006). To test this, a second female choice test was done between males whose immune system was challenged twice and control males that had not been subjected to any known immune challenges. It was predicted that treatment males should become more attractive to females than the control ones. During the third experiment, filaments were implanted for the third time to test whether terminal investment in reproduction was consistent (Hamilton and Zuk 1982). It was predicted that melanotic responses against nylon implants should be less intense in attractive males, since insects are thought to be unable invest in their reproduction and immunity simultaneously.

## Materials and Methods

### Insects

The beetles (n = 312) used in the experiment were collected from natural populations in several barns in southeastern Latvia in 2007. The stock culture was maintained at the University of Daugavpils at 27 ± 2° C on bran mixed with wheat flour, fresh carrots, and apples. Pupae were removed from the culture on the day of pupation. They were weighed, and their sex was determined by examining genitalia on the eighth abdominal segment (Bhattacharya et al. 1970). The pupae and newly emerged adults were kept individually in 200 ml plastic containers filled with bran and wheat flour mix and with fresh carrot/apple pieces offered *ad libitum* at least twice a week. Individuals with visible abnormalities from the experiment were discarded. All of the trials were performed in 2008.

### First implantation and female choice trial

After giving the beetles fresh fruits/vegetables *ad libitum*, 10 day old males were weighed (n = 232; body weight 122 ± 16.3 mg, mean ± SD) and assigned them to weight-matched pairs (±3% mass, n = 116, Figure 1). The pair member that received the treatment (implantation vs control) was randomly chosen. Since the glandular production of the sex pheromones is known to reach effective levels by day 7 post-imaginal eclosion (Menon 1976), at 12 days after imaginai enclosion the males were subjected to an immune system challenge. After immobilizing both pair members on ice, a piece of sterile nylon monofilament (2 mm length, 0.18 mm diameter, scratched with sandpaper, knotted at one end) was inserted into the treatment male through its pleural membrane between the third and fourth abdominal sternite. The control individuals had a hole punched in their pleural membrane between the third and fourth abdominal sternite but were not implanted with the nylon monofilament (Figure 1). Sexual attractiveness of these control males is known to equal the attractiveness of males that have not been subjected to puncture of their pleural membrane (Kivleniece et al. 2010). For the next 3 hours, each beetle was kept individually in numbered, small cylindrical translucent plastic canisters (30 mm diameter, 50 mm height) at a constant room temperature (23 ± 0.5° C). After 3 hours, the knot was carefully grasped and the monofilament implant was removed from each treatment male. The removal of the insert imitated a successful activity of the insect's immune system in destroying or eliminating a parasite egg or larvae. The period of 3 hours was sufficient to reach maximum rates of individual variation in melanization rates at 23° C (see e.g. Rantala et al. 2002). The removed implants were stored and dried for later analysis. Two implants could not be removed, reducing our number of treatment males and matched pairs to n=114 (Figure 1).

Treatment males were allowed to recover from any acute effects from the nylon insert treatment for 70–72 hours. Following this, the ability of males to attract a mate was evaluated via a female preference test (e.g., Rantala et al. 2002). More than two males were not presented in a female preference test to reduce the possibility of impaired female choice caused by pheromone interference within the restricted space of the female choice arena (Figure 1), since pheromones of male mealworm beetles are highly volatile are not perceivable to observers (Vainikka et al. 2007). Randomly selected virgin females were used for the tests, never using the same female twice.

**Figure 2.**
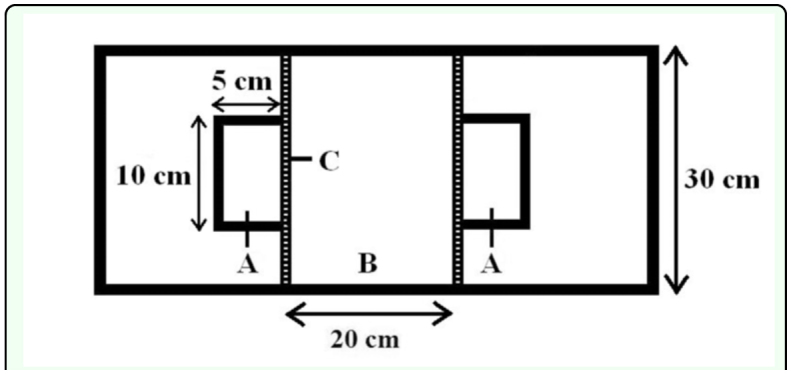
Female preference arena consisting of two smaller containers for the male individuals (A) and a larger container for the female (B) with the slots (C) in the sides of the female container. High quality figures are available online.

The dyadic preference arena consisted of a large container for the female and two smaller containers for the males that were attached outside opposite walls of the large container. The larger container was an open, topped, rectangular Plexiglas box, 20 by 30 cm, with a small section of 10 vertical slots the walls adjacent to the male containers attached to two opposite walls (Figure 2). The slots were just large enough for either beetle to insert its head into the adjacent container. The layer of bran on the floor was changed after every trial to exclude contamination of the bran with smell of the previous female. The plexiglas containers for males (10 by 5 cm area, 5 cm height) were open only on the side which shared the slots on the side of the female container (Figure 2). This arrangement allowed male pheromones to enter the female container and also allowed the males and female to make audible and tactile contact while remaining within their respective containers. The female container had an open top to prevent over saturation with male pheromones. The ventilation in the room was turned off to avoid dissemination of the pheromones by the air currents. In all tests air temperature was monitored in the female container, and the trials were carried out under the conditions of red light.

Each dyadic preference test (n = 114) was started by simultaneously placing the two (paired prior to the treatments, see above) males into the opposite male containers. After 1–2 min a virgin female beetle was placed in the center of the larger container but she was not released for another 3 min. After releasing female, the time it took for a female to choose a male was recorded as well as the specific activity behaviors of the male beetles. The criteria for a clear choice were when the female met and touched one of the males and remained in close contact for at least 2 min. The beetles were removed as soon a clear choice was made, and the experimental males were retained for the second implant treatment and female preference test. While a clear choice was usually confirmed within 2–6 min after the female was freed to move, there were five females that were considered to not have made any choice. Three of those dug in the bran layer after inspecting at least one male, one stayed motionless for 5 min after initially inspecting a male, and one dug in the bran layer without inspecting either male. We replaced these with fresh females, and each time promptly obtained a clear choice.

Since *T. molitor* males often chase their prospective mates, locomotor activity may be important to obtain matings in this species (Worden and Parker 2005). During the dyadic preference tests, male activity was scored according to five categories: (i) no activity = 0 points: male remained motionless; (ii) untargeted activity = 1 point: although male was active, the activity was mostly near its wall opposite from the female; (iii) weak activity = 2 points: male either spent up to 50% of time near slots and trying to put its head into the female container or the male spent 100% of time near the slots while making a few attempts to put its head into the female container; (iv) average response = 3 points: male spent up to 75% of time near slots trying to put its head into the female container; (v) strong activity = 4 points: male spent 100% of time near slots putting its head into the female container.

### Second implantation and female choice trial

At 120 hours after the onset of the first implant insertion treatment, the previously immune challenged males (n = 114) were subjected to a repeated implant treatment (Figure 1). After 70–72 hours another round of dyadic female preference tests was conducted for all of 114 repeatedly challenged males (Figure 1). The same group of unchallanged males was used as in the first female preference test as a control. The control individuals again had a hole punched in their pleural membrane between the third and fourth abdominal sternite but were not implanted with the nylon monofilament. The challenged and unchallenged males were combined in unique weight-matched combinations, but never using the two males in the same pair during female preference tests, and the treatment and control males were weight-matched again.

### A third implantation and survival of males

At 120 hours after the onset of the second (ca. 240 hours after the onset of the first immune challenge) activation of immune system in the treatment group, randomly chosen males (n = 36) that had already had two implants and were preferred by females during the second female choice trial, received an implant for the third time (Figure 1). This was done to see whether the males preferred by females had recovered their immune system while investing in terminal reproductive effort after the second immune challenge. Reduced levels of melanization of their inserts were predicted in the case of terminal reproductive investment. The rest of the twice challenged males (both preferred (n = 35) and not preferred by females (n = 43)), as well as all of the control group males (n = 114) were included in the survival group to compare survival rates of preferred and non-preferred males after the second immune challenge (Figure 1). Each male was kept individually in the 200 ml plastic containers for 30 days at a constant temperature (23 ± 2° C). It was predicted that the preferred males should suffer the increased mortality rates in the case of terminal reproductive investment, while the survival of non-preferred males should be equal to the survival rate of the control males

### Immune assays

To measure the strength of encapsulation response against nylon monofilament, the light reflectivity (grayscale value) of each nylon insert was analyzed after it had been thoroughly dried. To quantify light reflectivity each removed insert was photographed from three different directions under consistent light conditions using a Zeiss Lumar V12 Stereo microscope and Axio Cam MRc5 digital recorder. The digital images were then analyzed by using image analysis software (Image J). The area of that portion of the insert that had been within the beetle body was marked, and the program calculated the grayscale value. Since increasing melanization indicated a stronger immune response in this study, total black was used as a maximum possible encapsulation result.

**Figure 3.**
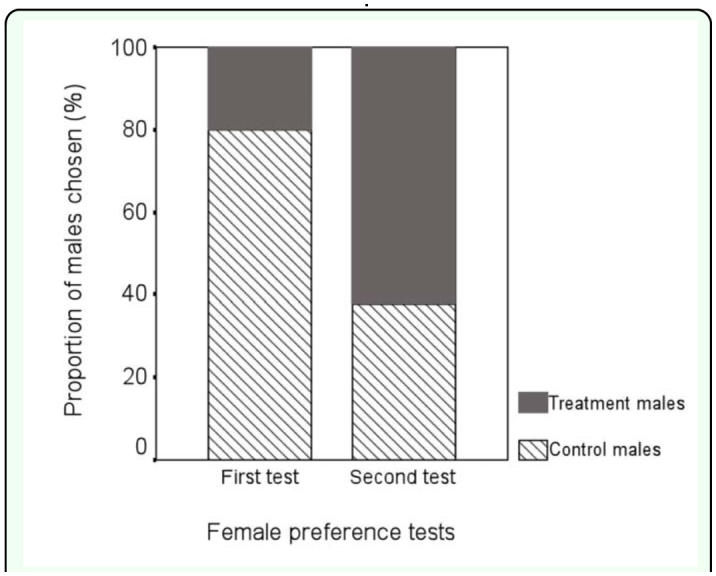
Female preference of immune-challenged male *Tenebrio molitor* after one and two immune challenges via nylon implantation events. High quality figures are available online.

### Statistics

Normality of the data was tested using one-sample Kolmogorov-Smirnov tests. Data on the time it took for a female to make a choice in preference tests as well as the rates of encapsulation response of the treatment males were normally distributed (all P values > 0.42), and parametric tests were used to analyze these variables. To examine time of female preference across the two trials, one-way ANOVA was used. Differences in encapsulation responses of the males across the three implants were analyzed using repeated measures ANOVA including the treatment as fixed factor. Probabilities from post hoc tests were calculated using Scheffé's test. Activity scores of males were not normally distributed, and we analyzed them by using non-parametric tests. Statistical analyses were performed with SPSS software (SPSS Inc., Chicago, Illinois).

## Results

**Figure 4.**
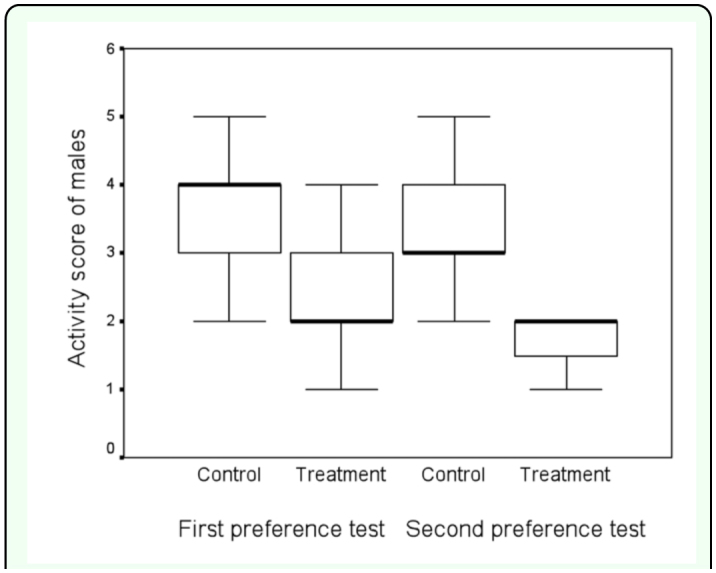
Box-and-whisker plot (thick bar = median, box = interquartile range, whiskers = full value range) of activity scores of males in control and treatment groups during the first and second female preference test. High quality figures are available online.

The first immune challenge by a nylon monofilament reduced the attractiveness of males. The females preferred control males in 91 out of 114 cases (2-tailed sign-test, z = -8.13, P < 0.001; Figure 3). On average, females made their choice in 186 ± 103 seconds (mean ± SD). Five females moved directly to their preferred male and stayed there. The rest of the females either first visited both males (n = 82) or inspected the other male from a distance of a few centimeters (n = 27) before staying with their preferred male. In the first female choice test, activity score of treatment males was lower than that of control males (2-tailed Mann-Whitney U-test: z = -8.36, n_1_ = n_2_ = 114, P < 0.001, Figure 4), suggesting that activation of immune response reduces male activity.

Following the second immune challenge, 71 out of 114 cases females preferred the treatment males over the control males (2-tailed sign-test: z = 5.00, P < 0.001; Figure 3). The females spent 170 ± 125 seconds (mean ± SD) to choose a preferred male in the second female preference test, and time to choose a male did not differ significantly between the first and second female preference tests (one-way ANOVA, F_1,28_ = 0.15, P = 0.70). Males with activated immune system had significantly lower activity scores than control males in the second female choice test (2-tailed Mann-Whitney U-test: z = -5.59, n_1_ = n_2_ = 114, P < 0.001; Figure 4). Treatment males decreased their activity during the second experimental phase in comparison to the first phase (2-tailed Wilcoxon's matched-pairs signed-ranks test: z = -4.29, n = 114, P < 0.001; Figure 4), while the activity of control males remained the same (2-tailed Wilcoxon's matched-pairs signed-ranks test: z = -1.85, n = 114, P = 0.07; Figure 4). Activity score of males preferred by females during the second female choice test was lower than activity of non-preferred males (2-tailed Mann-Whitney U-test: z = -6.27, n_1_ = 71, n_2_ = 43, P < 0.001), suggesting that locomotor activity may be traded-off against increased pheromonal attractiveness.

**Figure 5.**
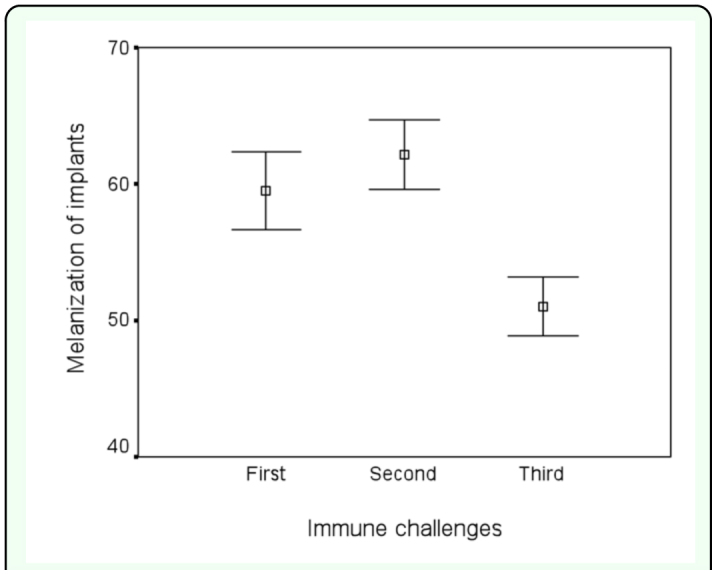
Mean encapsulation rates of treatment males after first, second, and third immune challenge. The grey-scale values of an implant was calibrated before the insertion to zero level indicating no melanization. Whiskers denote SE. High quality figures are available online.

The encapsulation response of the treatment males against the nylon monofilament was different across the treatment (repeated measures ANOVA, F_2_,_113_ = 191.21, P = 0.02, Figure 5). It was found to be significantly greater for the second immune challenge than for the first immune challenge (Scheffé's test P < 0.05, Figure 5), indicating that males, in general, invested in their immune system after the first challenge. After the first implantation, the encapsulation response of 23 treatment males preferred by females in the first female choice was marginally lower compared to the encapsulation rate of 91 treatment males non-preferred by females (2-tailed t-test: t = -1.26, n_1_ = 23, n_2_ = 91, P = 0.05), suggesting that some males may already have followed a terminal reproductive investment strategy after receiving their first implant. Moreover, the encapsulation rate of males preferred by females after the second implantation was significantly lower than that of the non-preferred males (2-tailed t-test: t = 0.16, n_1_ = 71, n_2_ = 43, P = 0.004).

35 out of 71 treatment males that were preferred by females in the second female choice test were assigned to the survival group. The rest of 36 treatment males preferred by females in the second preference test received the nylon monofilament for the third time. The encapsulation response was substantially lower for the third immune challenge in the preferred treatment males (Figure 5). It was found that the encapsulation response after the third implantation was significantly lower than the response for the first immune challenge of the same individuals (Scheffé's test P < 0.05, Figure 5), showing that the males did not make attempts to boost their immune systemafter the second activation of immune system.

Discounting the two initial deaths where implants could not be removed, all 114 treatment males survived for 6 days after the first implantation. In the survival group of the treatment males preferred by females in the second female choice test, all of 35 males (100%) died during the month following the second treatment, while only 10 out of 43 (23.26%) treatment males not preferred by females in the second female preference test died during the same period (2-tailed sign-test, z = -5.00, P < 0.001). Only 7 out of 114 control males died (7.98%) during the following month after the second immune challenge.

## Discussion

The results of the first female preference test showed that the attractiveness and activity score of the majority of treatment males decreased after they received their first implants. After removal of the inserts, the majority of these males appeared to invest in the recovery of their immune system as evidenced by their greater encapsulation responses during the second implantation, while some of the males seemed to have already chosen a terminal reproductive investment strategy. Overall, this indicates that the treatment males were not able to allocate resources simultaneously to both improvement of their health and to the increase of their sexual attractiveness (Ahtiainen et al. 2005). This shows that health of *T.molitor* is condition-dependent, suggesting that the immune system in this species can be easily impaired by physiological stress (Suwanchaichinda and Paskewitz 1998; Siva-Jothy and Thompson 2002; Rantala et al. 2003a; Yang et al. 2007). Our results suggest a trade-off between immunity and sexual attractiveness (Zahavi 1975; Sheldon and Verhulst 1996; Møller et al. 1999), supporting many other studies on animals (Saino et al. 1999; Jacot et al. 2004; Mougeot et al. 2004; Kilpimaa et al. 2004; Peters et al. 2004; Simmons et al. 2005; Pomfret and Knell 2006; Zala et al. 2008). The recovery of the immune system found in our study may be considered also as individual immune priming — a lasting, improved response after an initial exposure that deserves the future research in *T.molitor* (Moret and Siva-Jothy 2003; Jacot et al. 2005; Moret 2006; Sadd and Schmid-Hempel 2009).

The second female choice test also supported a trade-off between immunity and sexual signaling. This test revealed that most of the males subjected to a repeated immune challenge became more attractive to females than the unchallenged individuals, while the encapsulation rate of the treatment males significantly decreased after the second immune challenge as evidenced by the encapsulation rates of their third implants. This change of encapsulation response coincided with a decreased activity score and a significant change of female choice. This result suggests terminal investment on sexual signaling based on a trade-off between pheromone production and energy expenditures for other needs, such as locomotor activity and recovery of immunity. In addition to serving as mate attractors, sex pheromones also relay important information to the prospective mates (August, 1971; Hurd & Parry, 1991). It has been shown that pheromone production is costly and that female mealworm beetles prefer pheromones from males with better immunocompetence, indicated by a faster encapsulation rate against a novel antigen, and higher levels of phenoloxidase in haemolymph (Rantala et al. 2002, 2003). A trade-off between pheromone production and immunity in males is indirectly supported by the fact that females consistently preferred males that invested significantly less in the recovery of their immune system as indicated by encapsulation rate of their third implants. In our study the interplay between production of pheromones and condition of immune system in the treatment and control males was not measured. However, male signaling under conditions of terminal investment should be pheromone-related, and female preferences need to be studied in the future research by using male pheromone samples (Vainikka et al. 2007).

Increasing evidence shows that the immune response to a nylon implant is costly to mealworm beetles both in the context of short-term resource requirements (Siva-Jothy and Thompson 2002; Rantala et al. 2003a) as well as in a long term life-history perspective (Sadd et al. 2006). The hypothesis of terminal investment predicts that a host suffering from an infection that negatively affects its survival probability should allocate its resources towards immediate reproduction. In our study the treatment males responded to the repeated parasite-like haemocelic immune challenge as a survivorship threat (Sadd et al. 2006; Creighton et al. 2009), and they reduced the cost of ‘parasitism’ by increasing resource allocation towards sexual signaling in a last attempt to increase their individual fitness. Such effects were not found when treatment beetles received their implants for the first time since the short-term implantation was not followed by the decreased survival. However, after a repeated exposure to physiological stress caused by a synthetic parasite the beetles decreased their activity score and reduced the encapsulation rates. Thus the treatment males boosted the investment in sexual attractiveness at the expense of their immune system and locomotory ability. Since male mealworm beetles are able to rapidly modulate their attractiveness traits (Sadd et al. 2006), the outcome of immunity—reproduction interplay may be strongly dependent on the future reproductive value, which should be taken into account when evaluating parasite-mediated sexual selection (Hamilton and Zuk 1982).
